# Associations of body mass index and chemotherapy emetogenic risk with sleep disturbance in patients with cancer: a retrospective study

**DOI:** 10.3389/fpsyt.2026.1902262

**Published:** 2026-08-21

**Authors:** Chunlin Feng, Jun Dai, Jingju Liu, Ling Zhu, Zhang Zhang, Qiming Sun

**Affiliations:** 1 Department of Oncology, The Second People’s Hospital, Wuhu, Wuhu, Anhui, China; 2 Department of Nursing, The Second People’s Hospital, Wuhu, Wuhu, Anhui, China; 3 Outpatient Department, The Second People’s Hospital, Wuhu, Wuhu, Anhui, China; 4 Department of Endocrinology, The Second People’s Hospital, Wuhu, Wuhu, Anhui, China

**Keywords:** body mass index, cancer, chemotherapy emetogenic risk, joint associations, sleep disturbance

## Abstract

**Objective:**

To examine the associations of body mass index (BMI) and chemotherapy emetogenic risk with sleep disturbance in patients with cancer and to explore their multiplicative interaction and joint associations.

**Methods:**

This retrospective cross-sectional study analyzed the medical records of 300 patients with cancer who received chemotherapy at Wuhu Second People’s Hospital between January 2023 and February 2025. Sleep quality was assessed using the Pittsburgh Sleep Quality Index (PSQI). Multivariable binary logistic regression models were used to examine the associations of BMI and chemotherapy emetogenic risk with sleep disturbance after adjustment for age, sex, cancer type, educational level, and marital status. Multiplicative interaction and joint-association analyses were conducted as exploratory analyses, using patients with normal weight receiving low-to-moderately emetogenic chemotherapy as the reference group in the joint-association analysis.

**Results:**

A total of 153 patients (51.0%) had sleep disturbance. After adjustment for age, sex, cancer type, educational level, and marital status, underweight patients (aOR = 2.272, 95% CI: 1.180–4.374, P = 0.014), overweight/obese patients (aOR = 3.171, 95% CI: 1.707–5.893, P < 0.001), and patients receiving highly emetogenic chemotherapy (aOR = 3.009, 95% CI: 1.819–4.977, P < 0.001) had higher odds of sleep disturbance. The overall multiplicative interaction between BMI category and chemotherapy emetogenic risk was statistically significant (P for interaction = 0.008). In the joint-association analysis, underweight patients receiving highly emetogenic chemotherapy had higher odds of sleep disturbance (aOR = 7.373, 95% CI: 2.644–20.555, P < 0.001), while overweight/obese patients receiving highly emetogenic chemotherapy showed the highest observed odds (aOR = 13.794, 95% CI: 4.740–40.139, P < 0.001); 87.2% of patients in this subgroup had sleep disturbance.

**Conclusions:**

Abnormal BMI and high chemotherapy emetogenic risk were associated with higher odds of sleep disturbance among patients with cancer. The exploratory interaction and joint-association analyses suggested that patients with abnormal BMI receiving highly emetogenic chemotherapy, particularly those with overweight/obesity, may represent a clinically vulnerable subgroup requiring closer sleep assessment. Given the retrospective cross-sectional design, relatively small subgroup sizes, and wide confidence intervals, these findings should be interpreted as exploratory and do not establish causal relationships.

## 1 Introduction

With global population aging and lifestyle changes, cancer remains a major public health challenge worldwide (1). Chemotherapy is a cornerstone of cancer treatment, but its adverse effects can substantially impair patients’ quality of life. Chemotherapy-induced nausea and vomiting (CINV) is among the most distressing treatment-related symptoms (2). Although modern antiemetic strategies have improved symptom control, patients receiving highly emetogenic chemotherapy may still experience considerable symptom burden (3).

Sleep disturbance is also common among patients with cancer, with more than half experiencing impaired sleep quality (4). Poor sleep may adversely affect daily functioning and psychological well-being and has been associated with inflammatory changes during oncological treatment (5). Experimental evidence further suggests that sleep deficiency and circadian disruption may influence cancer-related biological processes (6). However, evidence regarding the association between the emetogenic classification of chemotherapy regimens and sleep disturbance remains limited.

Body mass index (BMI) is a widely used indicator of nutritional and metabolic status. Short sleep duration has been associated with central obesity (7). An updated systematic review also documented an association between sleep duration and obesity in adults (8). In addition, short sleep duration has been associated with a range of adverse health outcomes (9). Thus, abnormal BMI may be relevant to sleep disturbance among patients undergoing chemotherapy.

Sleep disturbance in patients with cancer is multifactorial, and previous studies have examined sleep problems in relation to cancer treatment, BMI, and chemotherapy-induced nausea and vomiting (10–12). Given the high prevalence of sleep disturbance among patients with cancer (13), identifying potentially vulnerable subgroups is clinically relevant. Therefore, this retrospective cross-sectional study aimed to examine the associations of BMI and chemotherapy emetogenic risk with sleep disturbance among patients with cancer and to explore their multiplicative interaction and joint associations. These exploratory analyses were intended to identify clinically vulnerable subgroups that may benefit from closer sleep assessment and supportive care.

## 2 Materials and methods

### 2.1 Study design and participants

This retrospective cross-sectional study used existing medical records of patients with cancer who received chemotherapy at Wuhu Second People’s Hospital between January 2023 and February 2025. Patients were eligible if they met the following criteria: (1) pathologically confirmed malignancy; (2) receipt of chemotherapy; (3) completion of the Pittsburgh Sleep Quality Index (PSQI); and (4) availability of complete clinical data. Patients were excluded if they (1) had severe psychiatric disorders or cognitive impairment, or were unable to complete the assessment; (2) had severe cardiac, hepatic, renal, or other major organ dysfunction; or (3) had incomplete clinical data.

This study was approved by the Ethics Committee of Wuhu Second People’s Hospital (Approval No.: 2026-KY-045) and was conducted in accordance with the Declaration of Helsinki. Given the retrospective design and the use of de-identified clinical records, the requirement for written informed consent was waived by the Ethics Committee.

### 2.2 Data collection

Demographic and clinical data included age, sex, height, weight, marital status, educational level, cancer type, and chemotherapy emetogenic classification. Body mass index (BMI) was calculated as weight in kilograms divided by height in meters squared (kg/m²). According to the Chinese criteria for adults, participants were categorized as underweight (<18.5 kg/m²), normal weight (18.5–23.9 kg/m²), or overweight/obese (≥24.0 kg/m²) (14).

### 2.3 Assessment of chemotherapy emetogenic risk

The emetogenic risk of chemotherapy regimens was classified according to the Chinese Guidelines for the Prevention and Treatment of Antitumor Therapy-Induced Nausea and Vomiting (2023 edition) (15). Based on the risk of acute chemotherapy-induced nausea and vomiting (CINV), chemotherapy regimens were categorized into a high emetogenic risk group and a low-to-moderate emetogenic risk group. The latter included regimens with moderate, low, or minimal emetogenic potential.

### 2.4 Assessment of sleep quality

The PSQI was completed after hospital admission and before the scheduled chemotherapy administration during the index hospitalization. The questionnaire assessed sleep quality during the preceding month (16). The PSQI consists of 19 self-reported items and evaluates seven components: subjective sleep quality, sleep latency, sleep duration, habitual sleep efficiency, sleep disturbances, use of sleep medication, and daytime dysfunction. Total scores range from 0 to 21, with higher scores indicating poorer sleep quality. The Chinese version of the PSQI has demonstrated satisfactory reliability and validity, with a Cronbach’s α coefficient of 0.790 (17). In the present study, a PSQI score >7 was used to define sleep disturbance, whereas a score ≤7 indicated the absence of sleep disturbance (18).

### 2.5 Statistical analysis

All statistical analyses were performed using SPSS version 26.0 and R version 4.3.2. Categorical variables are presented as frequencies and percentages and were compared using the chi-square test, whereas normally distributed continuous variables are presented as mean ± standard deviation and were compared using the independent-samples t-test. Sleep disturbance was coded as 0 = no and 1 = yes. BMI category and chemotherapy emetogenic risk were included as the primary exposure variables in multivariable binary logistic regression models, with adjustment for age, sex, cancer type, educational level, and marital status. Multiplicative interaction was assessed by including product terms between BMI category and chemotherapy emetogenic risk in the regression model. Joint exposure categories were also examined, using patients with normal weight receiving low-to-moderately emetogenic chemotherapy as the reference group. Odds ratios (ORs) and adjusted odds ratios (aORs) with 95% confidence intervals (CIs) were reported. Model calibration was assessed using the Hosmer–Lemeshow goodness-of-fit test, and model discrimination was evaluated using receiver operating characteristic curve analysis and the area under the curve (AUC). Given the relatively small subgroup sizes and wide confidence intervals, the interaction and joint-association analyses were considered exploratory. The final analytic dataset contained complete information for all variables used in the analyses; therefore, no missing-data imputation was performed. All statistical tests were two-sided, with P < 0.05 considered statistically significant.

## 3 Results

### 3.1 Comparison of demographic and clinical characteristics

Among the 300 patients undergoing chemotherapy, 153 (51.0%) were identified as having sleep disturbances (PSQI > 7). Univariate analyses demonstrated significant associations between sleep disturbances and both BMI and the emetogenic risk of chemotherapy regimens (both P<0.05). Patients who were overweight or obese and those receiving highly emetogenic chemotherapy were more likely to report sleep disturbances.

In contrast, no statistically significant differences were found between the sleep disturbance and non-sleep disturbance groups with respect to sex, age, educational attainment, marital status, or tumor type (all P > 0.05). The detailed comparisons are shown in **Table 1**.

**Table 1:** Comparison of demographic and clinical characteristics between patients with and without sleep disturbances (n = 300).

Variable	Category	PSQI score ≤7(n=147)	PSQI score > 7(n=153)	*χ^2^/t*	*P*
Sex				0.005	0.944
	Male	83	87		
	Female	64	66		
Educational level				2.122	0.713
	Illiterate	35	40		
	Primary school	56	63		
	Junior high school	35	29		
	Senior high school	12	15		
	College degree or above	9	6		
Marital status				2.036	0.361
	Unmarried	4	7		
	Married	139	138		
	Divorced/Widowed	4	8		
Tumor type				3.963	0.265
	Digestive system tumors	68	91		
	Lung cancer	31	24		
	Breast and gynecological cancers	26	24		
	Other tumors	22	14		
BMI				17.408	<0.001
	Underweight	23	36		
	Normal weight	100	68		
	Overweight/Obese	24	49		
Emetogenic risk				18.261	<0.001
	Low-to-moderate emetogenic risk	92	58		
	High emetogenic risk	55	95		
Age (years)		64.70 ± 10.94	64.15 ± 11.14	-0.431	0.666

### 3.2 Multivariable logistic regression analysis of factors associated with sleep disturbance among patients with cancer

After adjustment for age, sex, cancer type, educational level, and marital status, BMI category and chemotherapy emetogenic risk remained significantly associated with sleep disturbance. The overall association between BMI category and sleep disturbance was statistically significant (P < 0.001). Compared with patients with normal weight, underweight patients had higher odds of sleep disturbance (aOR = 2.272, 95% CI: 1.180–4.374, P = 0.014), and overweight/obese patients also had higher odds of sleep disturbance (aOR = 3.171, 95% CI: 1.707–5.893, P < 0.001). Patients receiving highly emetogenic chemotherapy had higher odds of sleep disturbance than those receiving low-to-moderately emetogenic chemotherapy (aOR = 3.009, 95% CI: 1.819–4.977, P < 0.001). No statistically significant associations were observed for age, sex, cancer type, educational level, or marital status (all P > 0.05). The Hosmer–Lemeshow goodness-of-fit test was not statistically significant (χ² = 11.991, df = 8, P = 0.152), indicating no evidence of poor model fit. Receiver operating characteristic curve analysis yielded an AUC of 0.712 (95% CI: 0.654–0.769), indicating moderate discrimination. Detailed results are presented in **Table 2**.

**Table 2:** Multivariable binary logistic regression analysis of factors associated with sleep disturbance among patients with cancer.

Factor	B	SE	Wald χ²	P	aOR	95% CI
BMI category, overall	—	—	15.737	<0.001	—	—
Normal weight	—	—	—	—	1.000	Reference
Underweight	0.821	0.334	6.034	0.014	2.272	1.180–4.374
Overweight/obese	1.154	0.316	13.329	<0.001	3.171	1.707–5.893
Chemotherapy emetogenic risk						
Low-to-moderate risk	—	—	—	—	1.000	Reference
High risk	1.102	0.257	18.416	<0.001	3.009	1.819–4.977
Sex						
Female	—	—	—	—	1.000	Reference
Male	0.316	0.284	1.243	0.265	1.372	0.787–2.392
Age, per 1-year increase	−0.010	0.013	0.589	0.443	0.990	0.965–1.015
Cancer type, overall	—	—	6.526	0.089	—	—
Digestive system tumors	—	—	—	—	1.000	Reference
Lung cancer	−0.610	0.347	3.078	0.079	0.544	0.275–1.074
Breast and gynecological cancers	0.463	0.359	1.665	0.197	1.589	0.786–3.212
Other tumors	0.199	0.411	0.235	0.628	1.221	0.545–2.733
Educational level, overall	—	—	1.353	0.852	—	—
Illiterate	—	—	—	—	1.000	Reference
Primary school	−0.127	0.337	0.143	0.706	0.881	0.455–1.704
Junior high school	−0.406	0.408	0.990	0.320	0.666	0.300–1.482
Senior high school	−0.124	0.509	0.060	0.807	0.883	0.326–2.393
College degree or above	−0.462	0.623	0.549	0.459	0.630	0.186–2.138
Marital status, overall	—	—	1.886	0.389	—	—
Unmarried	—	—	—	—	1.000	Reference
Married	0.991	0.726	1.862	0.172	2.693	0.649–11.174
Divorced/widowed	1.058	0.955	1.226	0.268	2.880	0.443–18.727

aOR, adjusted odds ratio; CI, confidence interval; SE, standard error.

All variables listed in the table were entered simultaneously into the multivariable binary logistic regression model. Normal weight, low-to-moderate emetogenic risk, female sex, digestive system tumors, illiterate educational level, and unmarried status were used as the reference categories. Owing to small cell sizes, urinary system tumors, hematologic malignancies, and other tumors were combined into the “other tumors” category.

### 3.3 Exploratory multiplicative interaction between BMI category and chemotherapy emetogenic risk in relation to sleep disturbance

The exploratory multiplicative interaction analysis showed a statistically significant overall interaction between BMI category and chemotherapy emetogenic risk after adjustment for age, sex, cancer type, educational level, and marital status (P for interaction = 0.008). The interaction terms were statistically significant for both underweight patients (aOR = 4.128, 95% CI: 1.047–16.275, P = 0.043) and overweight/obese patients (aOR = 6.137, 95% CI: 1.591–23.672, P = 0.008). These findings suggest that BMI category may modify the association between chemotherapy emetogenic risk and sleep disturbance, with stronger associations observed among patients with underweight or overweight/obesity than among those with normal weight. However, given the relatively small subgroup sizes and wide confidence intervals, interaction findings should be interpreted as exploratory. Detailed results are presented in **Table 3**.

**Table 3:** Exploratory multiplicative interaction between BMI category and chemotherapy emetogenic risk in relation to sleep disturbance.

Variables	Crude model			Adjusted model		
	*OR*	95%*CI*	*P*	*aOR*	95%*CI*	*P*
Normal weight	1.000	Reference	—	1.000	Reference	—
Underweight	1.132	0.473–2.714	0.780	1.158	0.468–2.869	0.751
Overweight/obese	1.382	0.618–3.088	0.431	1.458	0.631–3.371	0.378
Low-to-moderate emetogenic risk	1.000	Reference	—	1.000	Reference	—
High emetogenic risk	1.432	0.771–2.658	0.255	1.542	0.805–2.953	0.192
Underweight × high emetogenic risk	4.497	1.197–16.896	0.026	4.128	1.047–16.275	0.043
Overweight/obese × high emetogenic risk	6.016	1.619–22.348	0.007	6.137	1.591–23.672	0.008

OR, odds ratio; aOR, adjusted odds ratio; CI, confidence interval.

Normal weight and low-to-moderate emetogenic risk were used as the reference categories. The adjusted model included age, sex, cancer type, educational level, and marital status. The overall interaction was evaluated by comparing the adjusted models with and without the interaction terms. Given the relatively small subgroup sizes and wide confidence intervals, the interaction analysis should be interpreted as exploratory.

### 3.4 Exploratory joint associations of body mass index and emetogenic risk with sleep disturbance among patients with cancer

Using patients with normal weight receiving low-to-moderately emetogenic chemotherapy as the reference group, underweight patients receiving highly emetogenic chemotherapy had markedly higher odds of sleep disturbance after adjustment for age, sex, cancer type, educational level, and marital status (aOR = 7.373, 95% CI: 2.644–20.555, P < 0.001). Overweight/obese patients receiving highly emetogenic chemotherapy showed the highest observed odds of sleep disturbance (aOR = 13.794, 95% CI: 4.740–40.139, P < 0.001), and 87.2% of patients in this subgroup experienced sleep disturbance. No statistically significant differences were observed for underweight patients receiving low-to-moderately emetogenic chemotherapy, normal-weight patients receiving highly emetogenic chemotherapy, or overweight/obese patients receiving low-to-moderately emetogenic chemotherapy. These findings suggest that patients with abnormal BMI who receive highly emetogenic chemotherapy, particularly those with overweight/obesity, may represent a clinically vulnerable subgroup for sleep disturbance. Given the relatively small subgroup sizes and wide confidence intervals, these joint associations should be interpreted as exploratory. Detailed results are presented in **Table 4**.

**Table 4:** Exploratory joint associations of BMI category and chemotherapy emetogenic risk with sleep disturbance.

BMI category	Emetogenic risk	n	PSQI score > 7, n (%)	Crude model		Adjusted model	
				OR (95%CI)	P	aOR (95%CI)	P
Normal weight	Low-to-moderate	88	32 (36.4)	1.000, Reference	—	1.000, Reference	—
Underweight	Low-to-moderate	28	11 (39.3)	1.132 (0.473–2.714)	0.780	1.158 (0.468–2.869)	0.751
Underweight	High	31	25 (80.6)	7.292 (2.706–19.648)	<0.001	7.373 (2.644–20.555)	<0.001
Normal weight	High	80	36 (45.0)	1.432 (0.771–2.658)	0.255	1.542 (0.805–2.953)	0.192
Overweight/obese	Low-to-moderate	34	15 (44.1)	1.382 (0.618–3.088)	0.431	1.458 (0.631–3.371)	0.378
Overweight/obese	High	39	34 (87.2)	11.900 (4.230–33.479)	<0.001	13.794 (4.740–40.139)	<0.001

OR, odds ratio; aOR, adjusted odds ratio; CI, confidence interval.

Patients with normal weight receiving low-to-moderately emetogenic chemotherapy were used as the reference group. The adjusted model included age, sex, cancer type, educational level, and marital status. Given the relatively small subgroup sizes and wide confidence intervals, the joint-association analysis should be interpreted as exploratory.

## 4 Discussion

In the present study, 300 patients undergoing chemotherapy were included, of whom 153 experienced sleep disturbances, yielding a prevalence of 51.0%. This finding is consistent with previous studies reporting that the prevalence of sleep disturbances among patients with cancer ranges from 40% to 70% (13, 19). Compared with the general population, patients with cancer are more vulnerable to sleep problems due to the combined effects of the disease itself, treatment-related adverse events, and psychosocial stressors (20). Symptoms commonly experienced during chemotherapy, such as nausea and vomiting, pain, and fatigue, may be associated with nocturnal sleep disruption through physical discomfort, psychological distress, circadian rhythm disruption, and neuroendocrine dysregulation. The present study further showed that abnormal BMI and high chemotherapy emetogenic risk were associated with higher odds of sleep disturbance after adjustment for age, sex, cancer type, educational level, and marital status. The exploratory interaction analysis indicated that the association between chemotherapy emetogenic risk and sleep disturbance differed across BMI categories. In the joint-association analysis, overweight/obese patients receiving highly emetogenic chemotherapy had the highest prevalence and adjusted odds of sleep disturbance. These findings suggest that considering BMI and chemotherapy emetogenic classification together may help identify patients who warrant closer sleep assessment.

The present study found that patients receiving highly emetogenic chemotherapy had higher odds of sleep disturbance than those receiving low-to-moderately emetogenic chemotherapy after adjustment for age, sex, cancer type, educational level, and marital status (aOR = 3.009, 95% CI: 1.819–4.977). This finding suggests an association between the emetogenic classification of chemotherapy regimens and sleep disturbance. Chemotherapy-induced nausea and vomiting (CINV) is one of the most common adverse effects of chemotherapy and an important contributor to reduced quality of life among patients with cancer (21). However, the emetogenic potential of a chemotherapy regimen is not equivalent to the actual occurrence or severity of nausea and vomiting. Patients receiving highly emetogenic chemotherapy may experience different symptom burdens depending on individual susceptibility and the effectiveness of antiemetic treatment. Because the actual frequency and severity of nausea and vomiting and the response to antiemetic therapy were not assessed in the present study, the observed association cannot be attributed to actual nausea and vomiting, and CINV cannot be considered a confirmed mediator.

Moreover, although the PSQI was completed after hospital admission and before the scheduled chemotherapy administration during the index hospitalization, it assessed sleep quality during the preceding month. Some patients may have received previous chemotherapy cycles before completing the PSQI. Therefore, the observed association cannot be attributed specifically to the chemotherapy administered during the index hospitalization, and the temporal sequence between chemotherapy emetogenic classification and sleep disturbance could not be definitively established.

Several factors may plausibly contribute to the observed association, although they were not directly assessed in the present study. Gastrointestinal discomfort, appetite loss, psychological distress, and circadian disruption may be related to poorer sleep during chemotherapy (22). In addition, certain chemotherapeutic agents and supportive medications, particularly corticosteroids and 5-HT3 receptor antagonists, may influence sleep through their effects on central nervous system function (3, 22). Because these factors were not directly assessed, they should be regarded as hypotheses for future investigation rather than mechanisms established by the present study. Nevertheless, the findings support closer assessment of sleep quality among patients receiving highly emetogenic chemotherapy regimens.

The present study demonstrated that abnormal BMI was associated with higher odds of sleep disturbance. Compared with patients with normal weight, underweight patients had higher odds of sleep disturbance (aOR = 2.272, 95% CI: 1.180–4.374), whereas overweight/obese patients had higher odds of sleep disturbance (aOR = 3.171, 95% CI: 1.707–5.893), after adjustment for age, sex, cancer type, educational level, and marital status. These findings suggest a potentially non-linear relationship between BMI category and sleep disturbance, with normal-weight patients showing the lowest observed odds in the present sample. Previous studies have reported a complex bidirectional association between BMI and sleep disturbance (23). On the one hand, overweight and obesity are often accompanied by chronic low-grade inflammation, insulin resistance, and neuroendocrine dysfunction. Elevated levels of inflammatory cytokines, such as interleukin-6 (IL-6) and tumor necrosis factor-α (TNF-α), may affect hypothalamic sleep-regulating centers, thereby altering sleep architecture and reducing sleep quality (24). In addition, obesity is strongly associated with obstructive sleep apnea, and recurrent nocturnal hypoxia and arousals may further exacerbate sleep fragmentation (25). On the other hand, underweight status and cancer-related cachexia may also adversely affect sleep through malnutrition, metabolic imbalance, and increased physiological consumption (26). Patients with cancer are particularly vulnerable to weight abnormalities because of disease progression, treatment-related adverse effects, and inadequate nutritional intake. The findings therefore support attention to both underweight and overweight/obese patients. However, the cross-sectional design does not allow the direction of the relationship between BMI and sleep disturbance to be determined.

The exploratory multiplicative interaction analysis indicated that the association between chemotherapy emetogenic risk and sleep disturbance differed according to BMI category. After adjustment for age, sex, cancer type, educational level, and marital status, the overall interaction was statistically significant (P for interaction = 0.008). The interaction terms were statistically significant for both underweight patients (aOR = 4.128, 95% CI: 1.047–16.275) and overweight/obese patients (aOR = 6.137, 95% CI: 1.591–23.672). These findings suggest that BMI may modify the association between chemotherapy emetogenic risk and sleep disturbance. Specifically, the association with high emetogenic risk was stronger among patients with abnormal BMI than among those with normal weight. This finding should not be interpreted as evidence that normal weight had a direct protective effect; rather, it indicates that the strength of the association differed across BMI categories.

The joint-association analysis provided a more clinically intuitive representation of this pattern. Using patients with normal weight receiving low-to-moderately emetogenic chemotherapy as the reference group, underweight patients receiving highly emetogenic chemotherapy had markedly higher odds of sleep disturbance (aOR = 7.373, 95% CI: 2.644–20.555). Overweight/obese patients receiving highly emetogenic chemotherapy had the highest observed odds of sleep disturbance (aOR = 13.794, 95% CI: 4.740–40.139), and the prevalence of sleep disturbance in this subgroup reached 87.2%.In contrast, underweight patients receiving low-to-moderately emetogenic chemotherapy, normal-weight patients receiving highly emetogenic chemotherapy, and overweight/obese patients receiving low-to-moderately emetogenic chemotherapy did not show statistically significant differences compared with the reference group. Taken together, these findings suggest that patients with abnormal BMI who receive highly emetogenic chemotherapy, particularly those with overweight/obesity, represent a clinically vulnerable subgroup for sleep disturbance.

Several hypotheses may help explain the observed pattern. Highly emetogenic chemotherapy regimens indicate a greater potential for treatment-related gastrointestinal symptoms, whereas abnormal BMI may reflect nutritional imbalance, altered metabolic status, inflammatory activity, or reduced physiological reserve (27). Sleep regulation is also related to inflammatory and neurobiological processes, including neurotrophic signaling (28). In addition, bidirectional communication between the gastrointestinal tract and the central nervous system may provide a broader theoretical framework for understanding the relationships among treatment-related gastrointestinal symptoms, nutritional status, and sleep (29). However, actual nausea and vomiting, inflammatory markers, neurotrophic factors, metabolic indicators, and gut–brain-related mechanisms were not directly assessed in the present study. These explanations should therefore be considered hypotheses for future research rather than mechanisms established by the current findings. From a clinical perspective, patients with abnormal BMI receiving highly emetogenic chemotherapy may warrant closer sleep assessment and individualized supportive care. Evidence-based sleep interventions, including cognitive behavioral therapy for insomnia, may be considered when clinically appropriate (30).

Several limitations of this study should be acknowledged. First, the single-center retrospective cross-sectional design limits the generalizability of the findings and precludes conclusions regarding causality or temporal relationships. The retrospective sampling process and the exclusion of patients with incomplete clinical data may also have introduced selection bias. Second, although the multivariable models were adjusted for age, sex, cancer type, educational level, and marital status, several clinically important determinants of sleep disturbance were not consistently available in the medical records. These included anxiety, depression, pain severity, fatigue, cancer stage, chemotherapy cycle, corticosteroid and antiemetic use, previous sleep disorders, obstructive sleep apnea, alcohol and smoking status, and sleep medication use. Detailed information on cancer stage, chemotherapy cycle number, and specific chemotherapy regimens could therefore not be comprehensively reported or included in the adjusted analyses. Residual confounding cannot be excluded. Third, chemotherapy emetogenic classification reflects the potential of a chemotherapy regimen to induce nausea and vomiting rather than the actual symptoms experienced by individual patients. The frequency and severity of chemotherapy-induced nausea and vomiting and the effectiveness of antiemetic treatment were not directly assessed. Moreover, although the PSQI was administered before the scheduled chemotherapy administration during the index hospitalization, it assessed sleep quality over the preceding month, during which some patients may have received previous chemotherapy cycles. Because chemotherapy cycle information was not consistently available, the temporal sequence between chemotherapy emetogenic classification and sleep disturbance could not be definitively established. Fourth, sleep disturbance was assessed using the PSQI, a subjective self-report measure, without objective sleep assessments such as actigraphy or polysomnography. In addition, BMI is a relatively crude indicator and does not directly reflect body composition, nutritional status, or cancer-related cachexia. Finally, several BMI–emetogenic risk subgroups included relatively small numbers of patients, resulting in wide confidence intervals and limited precision in the interaction and joint-association estimates. These analyses should therefore be interpreted as exploratory. Larger, multicenter prospective studies with more comprehensive clinical information and objective sleep assessments are needed to confirm these findings.

In conclusion, abnormal BMI and high chemotherapy emetogenic risk were each associated with higher odds of sleep disturbance among patients with cancer undergoing chemotherapy. Compared with patients with normal weight, both underweight and overweight/obese patients had higher odds of sleep disturbance. Exploratory interaction and joint-association analyses suggested that the association between chemotherapy emetogenic risk and sleep disturbance may differ across BMI categories. In particular, overweight/obese patients receiving highly emetogenic chemotherapy had the highest observed prevalence and odds of sleep disturbance. These findings suggest that patients with abnormal BMI receiving highly emetogenic chemotherapy may represent a clinically vulnerable subgroup who could benefit from closer sleep assessment, nutritional evaluation, and individualized supportive care. However, given the retrospective cross-sectional design, relatively small subgroup sizes, and wide confidence intervals, the interaction and joint-association findings should be interpreted as exploratory and require confirmation in larger prospective studies.

## Data Availability

The raw data supporting the conclusions of this article will be made available by the authors, without undue reservation.
